# Supplementary material for: Individual and collective gains from cooperation and reciprocity in a dynamic-network Prisoner’s Dilemma driven by extraversion, openness, and agreeableness
Source: Sci Rep. 2026 Apr 24;16:18926. doi: 10.1038/s41598-026-49942-w (PMC13276042; doi:10.1038/s41598-026-49942-w)
Supplement: Supplementary file 1 — Supplementary Information. [file 41598_2026_49942_MOESM1_ESM.pdf]

Supplementary Information for "Individual and collective gains from cooperation and reciprocity in a dynamic-network Prisoner's Dilemma driven by extraversion, openness, and agreeableness"

David Abián, Jorge Bernad, Sergio Ilarri, Raquel Trillo-Lado
Scientific Reports

Supplementary Note: Robustness for $\beta = 0$ and $\beta = 1$

To assess whether the main conclusions extend to the limiting decision rules, we added endpoint runs with $\beta = 0$ (maximally history-driven cooperation) and $\beta = 1$ (purely trait-driven cooperation, with no learning from partner-specific history). The main qualitative conclusions are unchanged and become sharper at these endpoints.

At $t = 300$, the Agreeableness–payoff association $\rho(A, \Pi)$ is positive for $\beta = 0$ in all 21 size×scenario conditions (range [0.435, 0.810]), but negative for $\beta = 1$ in all conditions (range [−0.941, −0.620]). Mean payoff per agent is higher at $\beta = 0$ than at $\beta = 1$ in every condition. In net-beneficial regimes (all scenarios except low-Agreeableness), $\beta = 0$ also yields lower harm prevalence and lower payoff inequality than $\beta = 1$ (Supplementary Table S1).

Degree assortativity remains near zero at both endpoints (maximum $|r| \approx 0.09$). Agreeableness assortativity is typically stronger at $\beta = 0$ than at $\beta = 1$, but it can remain near zero in uniformly cooperative high-Agreeableness populations under $\beta = 0$, consistent with rapid behavioural homogenisation under fully history-driven play.

Table S1: Robustness for $\beta = 0$ and $\beta = 1$, averaged across $N \in \{30, 100, 200\}$ (20 seeds per size).

	Payoff/agent		Harm (%)		Inequality I		$\rho(A, \Pi)$ at $t = 300$	
Scenario	$\beta = 0$	$\beta = 1$	$\beta = 0$	$\beta = 1$	$\beta = 0$	$\beta = 1$	$\beta = 0$	$\beta = 1$
balanced	813.8	321.5	1.7	14.0	0.286	0.474	0.707	−0.901
high-A	1553.4	873.9	0.0	0.0	0.160	0.190	0.490	−0.643
low-A	−160.2	−387.3	69.0	97.3	0.620	0.369	0.728	−0.836
high-E	1126.6	478.0	1.3	13.1	0.257	0.454	0.781	−0.938
low-E	493.5	178.7	2.5	15.7	0.337	0.500	0.626	−0.846
high-O	802.8	319.6	1.8	14.1	0.289	0.477	0.713	−0.903
low-O	814.7	330.4	1.5	13.6	0.290	0.467	0.705	−0.892

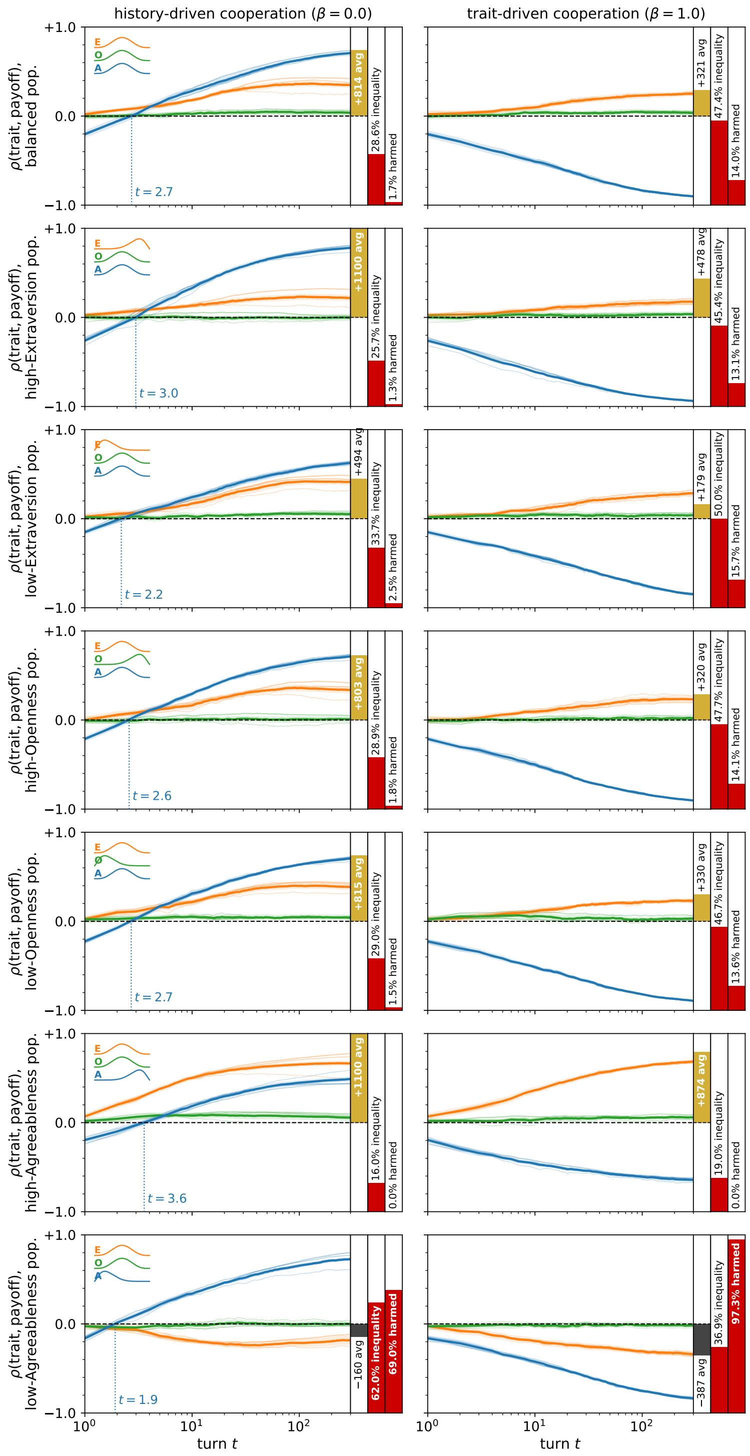

Figure S1: **Trait–payoff correlations over time across scenarios for the endpoint decision rules $\beta \in \{0, 1\}$.** Rows vary the population trait scenario; columns compare the two endpoint regimes. Within each panel, curves show the mean Spearman correlation between trait values and cumulative payoff over time, averaged across seeds and sizes, with uncertainty ribbons indicating 95% confidence intervals across seeds. The endpoint contrast sharpens the main result: when cooperation is fully history-driven ($\beta = 0$), Agreeableness becomes individually advantageous across all conditions, whereas under purely trait-driven behaviour ($\beta = 1$), higher Agreeableness remains individually costly throughout the horizon.

Supplementary Note: Sensitivity to the global damping parameter λ

To test whether the main conclusions depend on the baseline rewiring pace $\lambda = 0.5$, we reran the full main grid for $\lambda \in \{0.3, 0.7\}$ while keeping all other parameters unchanged. The qualitative picture is robust.

At $t = 300$, $\rho(A, \Pi)$ is positive in all 21 size×scenario conditions for $\beta = 0.2$ at both λ values (ranges [0.418, 0.821] and [0.509, 0.832] for $\lambda = 0.3$ and 0.7, respectively), and negative in all 21 conditions for $\beta = 0.8$ at both λ values (ranges [−0.820, −0.353] and [−0.843, −0.246]). For $\beta = 0.5$, the pattern is almost unchanged: $\rho(A, \Pi) > 0$ in all 21 conditions for $\lambda = 0.3$ and in 20 out of 21 for $\lambda = 0.7$; the only exception is the low-Extraversion, $N = 200$ condition, where the end-state estimate is effectively zero ($\rho = -0.007$ at $t = 300$).

The main effect of λ is on the *tempo* of rewiring rather than on the direction of the results. Averaged over all sizes, scenarios, and β values, $\lambda = 0.3$ yields mean network density 0.044 at $t = 1$, 0.069 at $t = 5$, and 0.072 at $t = 300$ (average degree $2.69 \to 4.36$), whereas $\lambda = 0.7$ yields density 0.071 at $t = 1$, 0.066 at $t = 5$, and 0.064 at $t = 300$ (average degree $4.29 \to 3.87$). Thus, larger λ produces a sharper early transient but a lower steady-state degree and density.

The clearest behavioural consequence is a delay in the point at which reciprocity makes Agreeableness individually advantageous. Averaging over all 21 size×scenario conditions, the first turn at which $\rho(A, \Pi) > 0$ shifts from 3.7 to 5.9 for $\beta = 0.2$. For $\beta = 0.5$, the corresponding mean shifts from 22.4 at $\lambda = 0.3$ to 78.9 among the 20 out of 21 conditions that cross at $\lambda = 0.7$; the remaining low-Extraversion, $N = 200$ condition does not cross by $H = 300$. As in the main text, no crossing occurs by $H = 300$ for $\beta = 0.8$.

End-state performance changes only moderately (Supplementary Table S2). Averaged across all 21 size×scenario conditions, $\lambda = 0.7$ slightly improves the history-dominant regime $\beta = 0.2$ (mean payoff/agent 396.7 vs. 383.7; harm 14.2% vs. 15.0%; inequality 0.259 vs. 0.282), while slightly lowering payoff in the balanced and trait-dominant regimes (310.1 vs. 320.6 for $\beta = 0.5$; 287.5 vs. 309.3 for $\beta = 0.8$), even as inequality is somewhat reduced. In hostile low-Agreeableness populations, larger λ is mildly protective because the lower steady-state degree reduces exposure (for example, averaged across N, payoff/agent improves from −409.1 to −348.3 at $\beta = 0.5$).

Degree assortativity remains near zero or slightly negative at both λ values (maximum $|r| \approx 0.084$ for $\lambda = 0.3$ and ≈ 0.105 for $\lambda = 0.7$), while Agreeableness assortativity persists in cooperative high-Agreeableness regimes and can be somewhat stronger for $\lambda = 0.7$ (maximum $r \approx 0.129$ vs. 0.212). Overall, moderate changes in λ affect convergence speed and steady-state exposure more than they affect the qualitative ordering of regimes. Supplementary Figs. S2 and S3 show the full time-series grids for $\lambda = 0.3$ and $\lambda = 0.7$, respectively.

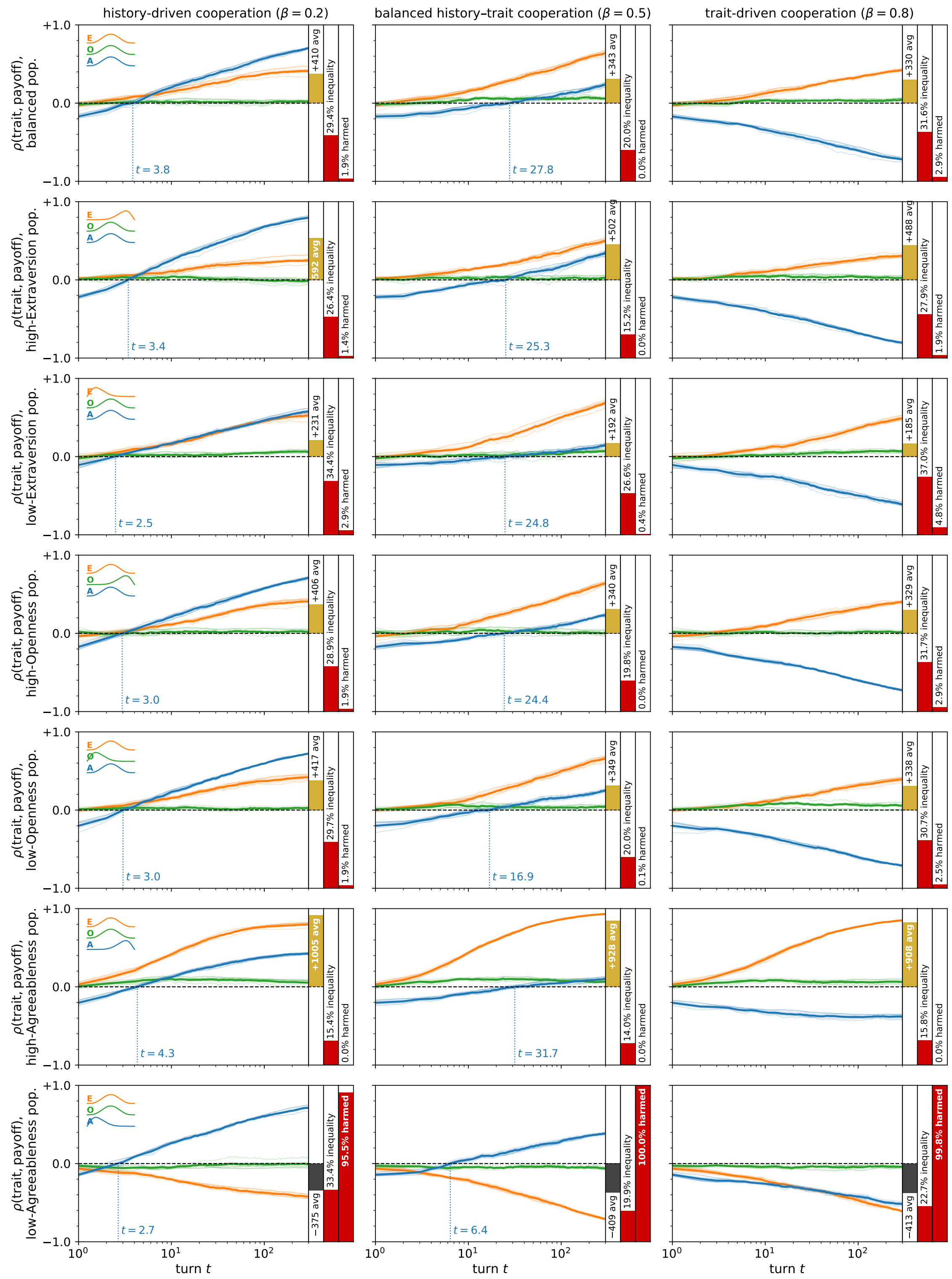

Figure S2: **Trait–payoff correlations over time across scenarios for $\lambda = 0.3$.** Rows vary the population trait scenario and columns vary the trait–history mixing weight β. Within each panel, curves show mean Spearman trait–payoff correlations over time, averaged across seeds and sizes, with 95% confidence intervals across seeds. Relative to the main setting $\lambda = 0.5$, the lower damping value produces slower early rewiring and a slightly denser steady state, but preserves the same qualitative ordering of regimes.

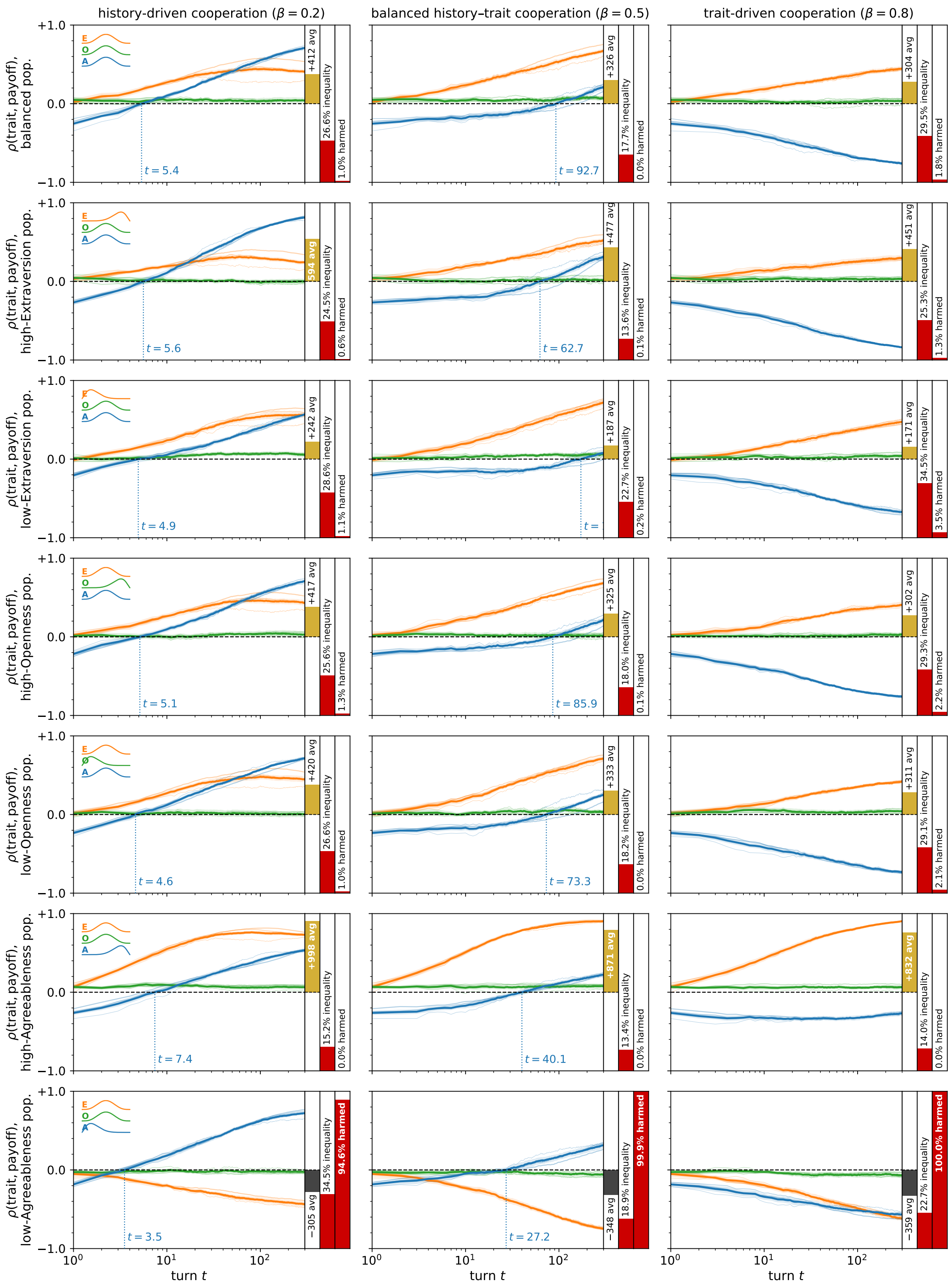

Figure S3: **Trait–payoff correlations over time across scenarios for $\lambda = 0.7$.** Rows vary the population trait scenario and columns vary the trait–history mixing weight β. Within each panel, curves show mean Spearman trait–payoff correlations over time, averaged across seeds and sizes, with 95% confidence intervals across seeds. Relative to the main setting $\lambda = 0.5$, the higher damping value produces a sharper early rewiring transient and a lower steady-state degree, while leaving the main sign patterns intact.

β	Scenario	Payoff/agent $\lambda = 0.3$	Payoff/agent $\lambda = 0.7$	Harm (%) $\lambda = 0.3$	Harm (%) $\lambda = 0.7$	Inequality I $\lambda = 0.3$	Inequality I $\lambda = 0.7$	$\rho(A, \Pi)$ at $t = 300$ $\lambda = 0.3$	$\rho(A, \Pi)$ at $t = 300$ $\lambda = 0.7$	First t, $\rho(A, \Pi) > 0$ $\lambda = 0.3$	First t, $\rho(A, \Pi) > 0$ $\lambda = 0.7$
0.2	balanced	410.2	411.6	1.9	1.0	0.294	0.266	0.701	0.710	4.3	6.0
	high-Agreeableness	1005.4	997.6	0.0	0.0	0.154	0.152	0.425	0.533	5.0	8.3
	low-Agreeableness	−375.1	−305.3	95.5	94.6	0.334	0.345	0.715	0.723	3.0	4.0
	high-Extraversion	592.1	594.2	1.4	0.6	0.264	0.245	0.794	0.813	3.7	6.0
	low-Extraversion	230.7	241.7	2.9	1.1	0.344	0.286	0.581	0.570	3.0	6.0
	high-Openness	406.2	417.1	1.9	1.3	0.289	0.256	0.709	0.709	3.3	5.3
	low-Openness	416.7	419.9	1.9	1.0	0.297	0.266	0.723	0.717	3.7	5.3
0.5	balanced	342.9	326.4	0.0	0.0	0.200	0.177	0.236	0.211	25.7	103.7
	high-Agreeableness	927.9	871.0	0.0	0.0	0.140	0.134	0.102	0.223	33.7	41.7
	low-Agreeableness	−409.1	−348.3	100.0	99.9	0.199	0.189	0.384	0.315	7.0	27.7
	high-Extraversion	501.8	477.0	0.0	0.1	0.152	0.136	0.339	0.315	22.7	73.0
	low-Extraversion	192.0	187.2	0.4	0.2	0.266	0.227	0.145	0.074	28.3	142.0*
	high-Openness	340.0	324.9	0.0	0.1	0.198	0.180	0.232	0.215	24.0	94.7
	low-Openness	348.9	332.8	0.1	0.0	0.200	0.182	0.251	0.254	15.3	90.7
0.8	balanced	329.6	304.3	2.9	1.8	0.316	0.295	−0.720	−0.760	$+\infty$	$+\infty$
	high-Agreeableness	908.4	832.5	0.0	0.0	0.158	0.140	−0.382	−0.268	$+\infty$	$+\infty$
	low-Agreeableness	−413.1	−359.4	99.8	100.0	0.227	0.227	−0.518	−0.567	$+\infty$	$+\infty$
	high-Extraversion	487.7	450.6	1.9	1.3	0.279	0.253	−0.803	−0.838	$+\infty$	$+\infty$
	low-Extraversion	185.5	171.2	4.8	3.5	0.370	0.345	−0.609	−0.671	$+\infty$	$+\infty$
	high-Openness	328.9	302.1	2.9	2.2	0.317	0.293	−0.727	−0.759	$+\infty$	$+\infty$
	low-Openness	338.2	311.0	2.5	2.1	0.307	0.291	−0.707	−0.730	$+\infty$	$+\infty$

Table S2: Sensitivity to the global damping parameter λ, averaged across $N \in \{30, 100, 200\}$ (20 seeds per size). The last two columns report the first turn at which the mean Agreeableness–payoff correlation becomes positive; $+\infty$ indicates that no crossing occurs by $H = 300$. *For $\beta = 0.5$, $\lambda = 0.7$, low-Extraversion, one of the three sizes ($N = 200$) does not cross by $H = 300$; the tabulated value averages the two sizes that do cross.
